# Toward a paradox of resources: the role of supportive climate and self-efficacy in the relationship between cynicism and intellectual engagement

**DOI:** 10.3389/fpsyg.2026.1718200

**Published:** 2026-07-09

**Authors:** Eliana Quiroz-González, Carmen Salvador-Ferrer, Pedro Antonio Díaz-Fúnez, Miguel Ángel Mañas-Rodríguez

**Affiliations:** 1 Psychology Program, Universidad Católica de Pereira, Pereira, Colombia; 2 IPTORA Research Team, Universidad de Almería, Almería, Spain

**Keywords:** cynicism, engagement, paradox, personal resources, self-efficacy, supportive climate

## Abstract

**Background:**

Research interest in intellectual engagement is increasing. The objective of this study was to examine the influence of cynicism on intellectual engagement through the mediation of supportive climate and the moderation of self-efficacy.

**Methods:**

The sample consisted of 336 Colombian workers who completed the MBI, the FOCUS-93, the ISA Engagement Scale, and the IPSICAP-24. Data were analyzed using descriptive statistics, Pearson’s correlation coefficient, and regression analysis with PROCESS Model 7.

**Results:**

The indirect effect of cynicism on intellectual engagement through supportive climate was statistically significant. Theps moderated mediation index was significant (index = −0.020, 95% CI [−0.047, −0.002]), confirming that the intensity of the indirect effect of cynicism on intellectual engagement through supportive climate depends on the levels of self-efficacy.

**Conclusion:**

As self-efficacy increases, the effect of cynicism on intellectual engagement through perceived supportive climate becomes more negative. This finding offers a critical perspective on psychological resources, exploratorily framed within what has been referred to as “the resources paradox”. The implications of these findings are discussed within the framework of organizational psychology.

## Introduction

Research interest in intellectual engagement has grown considerably in recent years (Abu-Hamour, 2018; Razzak et al., 2022; Azila-Gbettor, 2024; Kahil, 2025). Intellectual engagement is defined as the degree of intellectual absorption in work, characterized by concentration, cognitive effort, and sustained attention toward work-related tasks (Soane et al., 2012). Intellectual engagement is associated with greater task performance, organizational citizenship behavior (Soane et al., 2012), work-life balance (Nazneen, 2023) and performance (Díaz-Fúnez et al., 2021). Likewise, it is linked to lower turnover intention (Tauetsile, 2019) and role overload (Martín-Martín et al., 2022).

It is worth noting that certain psychosocial conditions can affect intellectual engagement, among them depersonalization or cynical attitudes (Mañas-Rodríguez et al., 2016). In particular, cynicism refers to a negative, insensitive, and detached response to different aspects of work (Maslach and Leiter, 2008; Maslach, 2017), which is associated with greater burnout (Kasemaa and Säälik, 2025). However, one factor that contributes to reducing cynicism is the perception of support (Sen et al., 2022).

In this vein, supportive climate refers to workers’ perception of the support they receive from coworkers, supervisors, or other areas of the organization (Luthans et al., 2008). Studies indicate that a supportive organizational environment facilitates organizational change processes, buffers the exhaustion generated by psychosocial risks, and reduces personal strain (Ferreira et al., 2018; Hayat and Afshari, 2020; Villavicencio-Ayub et al., 2022). In addition, perceived social support increases self-efficacy (Chiang et al., 2005; Kwon, 2018; Khan et al., 2022; Vilariño del Castillo and Lopez-Zafra, 2022; Dong et al., 2023; Chen et al., 2025; Liu et al., 2025).

Self-efficacy refers to the belief in one’s own capabilities to achieve a specific goal (Benight and Bandura, 2004). This psychological resource is related to confidence in facing challenging demands (Luthans and Youssef-Morgan, 2017) and creating positive spirals (Mañas-Rodríguez et al., 2020). Furthermore, it is negatively associated with anxiety and depression (Lu et al., 2023). A recent study reported that self-efficacy mediates the relationship between supportive climate and dedication (Quiroz-González et al., 2025). Additionally, self-efficacy has been recognized as a key construct within positive organizational psychology, given its contribution to understanding protective variables that foster employee well-being (Salanova et al., 2019), and has been identified as a moderator in the relationship between cynicism and work-related outcomes (Nazir et al., 2022). Research indicates that self-efficacy exerts moderating effects (Chen et al., 2025; Lahti et al., 2025) and functions as a protective resource (Bu et al., 2021; Edokpolor et al., 2024).

However, some studies highlight both the bright and dark sides of self-efficacy (Del Líbano et al., 2012). In some contexts, self-efficacy may lead to overconfidence, increasing the likelihood of errors (Vancouver et al., 2002), and negatively affecting motivation and performance quality (Vancouver et al., 2014), suggesting that its moderating role may be more complex than traditionally assumed. The specific work environment plays an important role in determining whether the consequences of self-efficacy are positive or negative (Salanova et al., 2012b; Uy et al., 2024). Given this complexity, self-efficacy occupies a central role in several theoretical models (Bandura, 1994; Salanova et al., 2012a; Hobfoll et al., 2018), including the Job Demands–Resources (JD-R) theory (Bakker and Demerouti, 2014, 2016).

The JD-R theory is perhaps the most influential framework for explaining workers’ well-being (Bakker et al., 2023). This theory of job design conceptualizes demands as the physical, psychological, and social aspects of work that require effort. In turn, resources have motivational potential: they facilitate goal achievement while simultaneously fostering development, promoting learning, and buffering the impact of demands. The relationship between job demands and resources affects both distress (health deterioration) and well-being (motivational process). In line with this motivational process, a meta-analysis addresses engagement from the JD-R theory perspective (Mazzetti et al., 2021).

The literature indicates that while cynicism (which is triggered by high demands and scarce resources) reduces engagement (a positive outcome) (Lehtiniemi et al., 2024), job resources (e.g., supportive climate) and personal resources (e.g., self-efficacy) (Bakker et al., 2023) may mitigate the impact of demands and foster engagement (Bakker and Demerouti, 2024). In this regard, social support has been reported to improve engagement (Torrente et al., 2012; Li et al., 2024; Ren et al., 2024; Murray et al., 2025). Therefore, supportive climate may play a mediating role in the relationship between cynicism and engagement.

**Figure 1:**
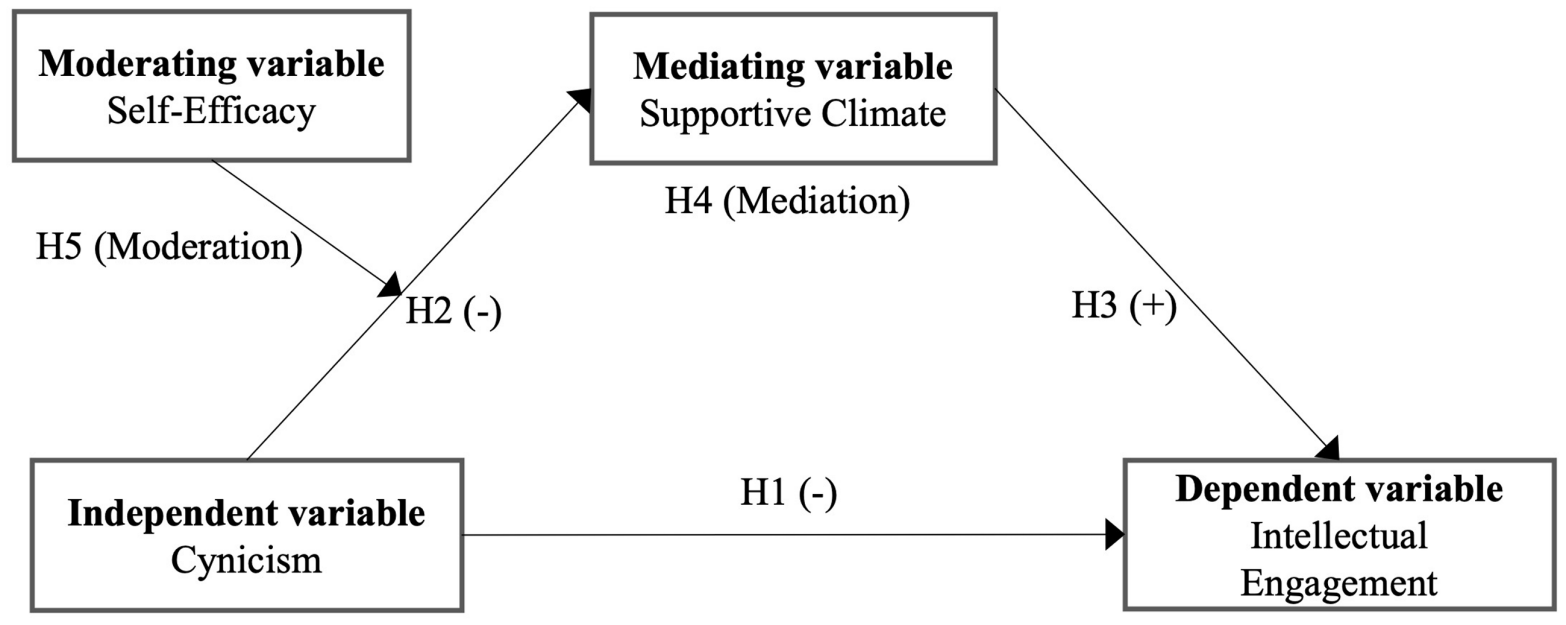
Predictive research model.

Although there is evidence supporting the role of supportive climate in various work contexts, the results are not entirely clear. Available evidence suggests that cynical attitudes reduce individuals’ willingness to invest cognitive effort in demanding tasks. However, the presence of contextual variables such as supportive climate and individual variables such as self-efficacy could significantly modify this relationship. Based on the above, the objective of this study is to examine the influence of cynicism on intellectual engagement through the mediation of supportive climate and the moderation of self-efficacy (see Figure 1).

As a consequence, the following hypotheses are proposed:

*H_1_*: Cynicism negatively predicts workers’ intellectual engagement.

*H_2_*: Cynicism negatively predicts supportive climate.

*H_3_*: Supportive climate positively predicts intellectual engagement.

*H_4_*: The relationship between cynicism and intellectual engagement is mediated by supportive climate.

*H_5_*: The relationship between cynicism and supportive climate is moderated by self-efficacy.

Understanding these dynamics expands the knowledge of these variables in organizational and work psychology and provides practical implications for the design of job positions that promote health in workplace contexts.

## Materials and methods

This research employed an associative strategy and an explanatory, cross-sectional design (Ato et al., 2013), with non-probabilistic sampling. Regarding inclusion criteria, Colombian workers who were of legal age and had an active employment relationship were considered. The sample consisted of 336 participants from different cities in Colombia, with an average age of 37. The characterization of the participants is presented in Table 1.

**Table 1:** Sociodemographic and work characteristics of the participants.

Characteristic	*n*	*%*
Sex		
Female	177	52.68
Male	159	47.32
Level of education		
High school	54	16.07
Technical or technological	58	17.26
Undergraduate degree	224	66.67
Marital status		
Married	99	29.46
Single	141	41.96
Divorced	13	3.87
Widowed	1	0.30
Common-law union	82	24.40
Type of organization		
Public	208	61.90
Private	128	38.10
Work sector		
Health	60	17.86
Education	86	25.60
Correctional system	125	37.20
Other services	65	19.35
Type of contract		
Fixed-term	73	21.73
Indefinite-term	182	54.17
Service provision	60	17.86
Work or labor	8	2.38
Apprenticeship	13	3.87
Job tenure		
1–5 years	160	47.60
6–10 years	70	20.80
11–15 years	48	14.30
16–20 years	21	6.30
More than 20 years	37	11.0

In the Colombian educational and labor context, educational attainment levels are defined as follows: a high school diploma indicates completion of secondary education; technical or technological studies refer to short-cycle post-secondary vocational programs; and an undergraduate degree corresponds to the completion of a university bachelor’s program. Regarding contract types, fixed-term contracts define employment for a set period, while indefinite-term contracts have no predetermined end date; service provision contracts involve an individual performing a specific, independent task without establishing an employer-employee relationship; work or labor contracts are tied to the completion of a particular task or project, and apprenticeship contracts are training agreements intended for vocational education.

### Instruments

The Maslach Burnout Inventory–General Survey, MBI-GS (Maslach et al., 1997) was used to assess cynicism based on items 8, 9, 13, 14, and 15 (e.g., “I doubt the value of my work”). The MBI is answered on a Likert-type scale with response options ranging from 0 = never to 6 = every day. Adequate psychometric properties have been reported in Colombia (Bravo et al., 2021). The cynicism subscale yielded satisfactory internal consistency in the present sample, with McDonald’s *ω* = 0.841 (95% CI [0.814, 0.868]) and Cronbach’s *α* = 0.843 (95% CI [0.813, 0.872]).

The ISA Engagement Scale (Soane et al., 2012) was used to assess intellectual engagement with items 1, 2, and 3 (e.g., “I pay a lot of attention to my work”). This scale uses a 7-point Likert-type response format (1 = strongly disagree and 7 = strongly agree). In this study, the Spanish validation was used (Mañas-Rodríguez et al., 2016). In the present sample, the intellectual engagement subscale showed excellent reliability, with McDonald’s *ω* = 0.933 (95% CI [0.921, 0.946]) and Cronbach’s *α* = 0.933 (95% CI [0.910, 0.956]).

The First Organizational Climate/Culture Unified Search, FOCUS-93 (Van Muijen and Koopman, 1999) was used to measure supportive climate through items 1, 2, 3, and 4 (e.g., “In my work area, colleagues help each other to get the work done”). This Likert-type format has response options ranging from 0 to 6, where 0 = never and 6 = always. In this study, FOCUS was used in its Colombian adaptation (García-Rubiano et al., 2025). The supportive climate subscale of the FOCUS yielded adequate internal consistency in the present sample, with McDonald’s ω = 0.860 (95% CI [0.836, 0.884]) and Cronbach’s α = 0.844 (95% CI [0.813, 0.875]).

Finally, the Colombian instrument for assessing psychological capital in organizations, IPSICAP-24 (Delgado-Abella and Mañas, 2019) was used to measure self-efficacy with items 21, 22, 23, and 24 (e.g., “I get involved in tasks that represent a challenge for me”). This questionnaire is answered on a 6-point Likert-type scale (1 = strongly disagree and 6 = strongly agree). Reliability estimates for the self-efficacy subscale of the IPSICAP were excellent in the present study, with McDonald’s ω = 0.935 (95% CI [0.925, 0.946]) and Cronbach’s α = 0.936 (95% CI [0.926, 0.946]).

### Procedure and data analysis

Data collection was conducted through an online Google Forms® questionnaire distributed via digital platforms and in-person contact. Completing the form took approximately 20 min. Subsequently, data analysis began with the calculation of linear correlations using Pearson’s coefficient (r) among the variables cynicism, intellectual engagement, supportive climate, and self-efficacy. A significance level of *p* < 0.05 was assumed.

In addition, moderated mediation analyses were conducted using a nonparametric bootstrapping procedure that estimated the direct and indirect influence of the independent variable on the dependent variable. For this purpose, Model 7 of the PROCESS macro version 4.2 (Hayes, 2022) was used with 10,000 bootstrap samples for 95% confidence intervals. Continuous predictors were mean-centered before creating interaction terms. Conditional effects were examined at low (−1 SD), mean, and high (+1 SD) levels of the moderator. To assess common method bias, Harman’s single-factor test was conducted including all study items in an unrotated exploratory factor analysis. The first factor accounted for 31.4% of the total variance, which is below the recommended threshold of 50%, suggesting that common method bias was unlikely to substantially affect the results. Furthermore, procedural remedies were implemented, including anonymous participation, confidentiality assurances, and the use of validated scales. The indirect and conditional influence was considered significant when the 95% confidence intervals (ICs) obtained through bias-corrected bootstrapping (BC), based on 10,000 samples, did not include zero.

### Ethical considerations

The study was conducted in accordance with the guidelines of the Universal Declaration of Ethical Principles for Psychologists (International Union of Psychological Science, 2008) and Law 1,090 of 2006 (Congreso de la República de Colombia, 2006). Participation was voluntary, and all participants provided informed consent. Likewise, the Ethics Committee of the Catholic University of Pereira approved this project (Minutes No. 007 of 2025).

## Results

As shown in Table 2, cynicism was negatively and significantly correlated with intellectual engagement (*r* = −0.25, *p* = 0.001), supportive climate (*r* = −0.39, *p* = 0.001), and self-efficacy (*r* = −0.15, *p* = 0.01). In turn, intellectual engagement was positively and significantly correlated with supportive climate (*r* = 0.20, *p* = 0.001) and self-efficacy (*r* = 0.27, *p* = 0.001). Finally, supportive climate was positively and significantly correlated with self-efficacy (*r* = 0.19, *p* = 0.001).

**Table 2:** Descriptive statistics and correlations for the study variables.

Variables	M	SD	1	2	3	4
1 Cynicism	2.07	1.60	–			
2 Intellectual engagement	5.59	1.25	−0.25^***^	–		
3 Supportive climate	3.67	1.53	−0.39^***^	0.20^***^	–	
4 Self-efficacy	4.63	0.85	−0.15^**^	0.27^***^	0.19^***^	–

**p < 0.01, ***p < 0.001. 95% confidence intervals: cynicism–intellectual engagement [−0.345, −0.144]; cynicism–supportive climate [−0.474, −0.292]; cynicism–self-efficacy [−0.256, −0.047]; intellectual engagement–supportive climate [0.095, 0.301]; intellectual engagement–self-efficacy [0.166, 0.364]; supportive climate–self-efficacy [0.088, 0.294].

**Figure 2:**
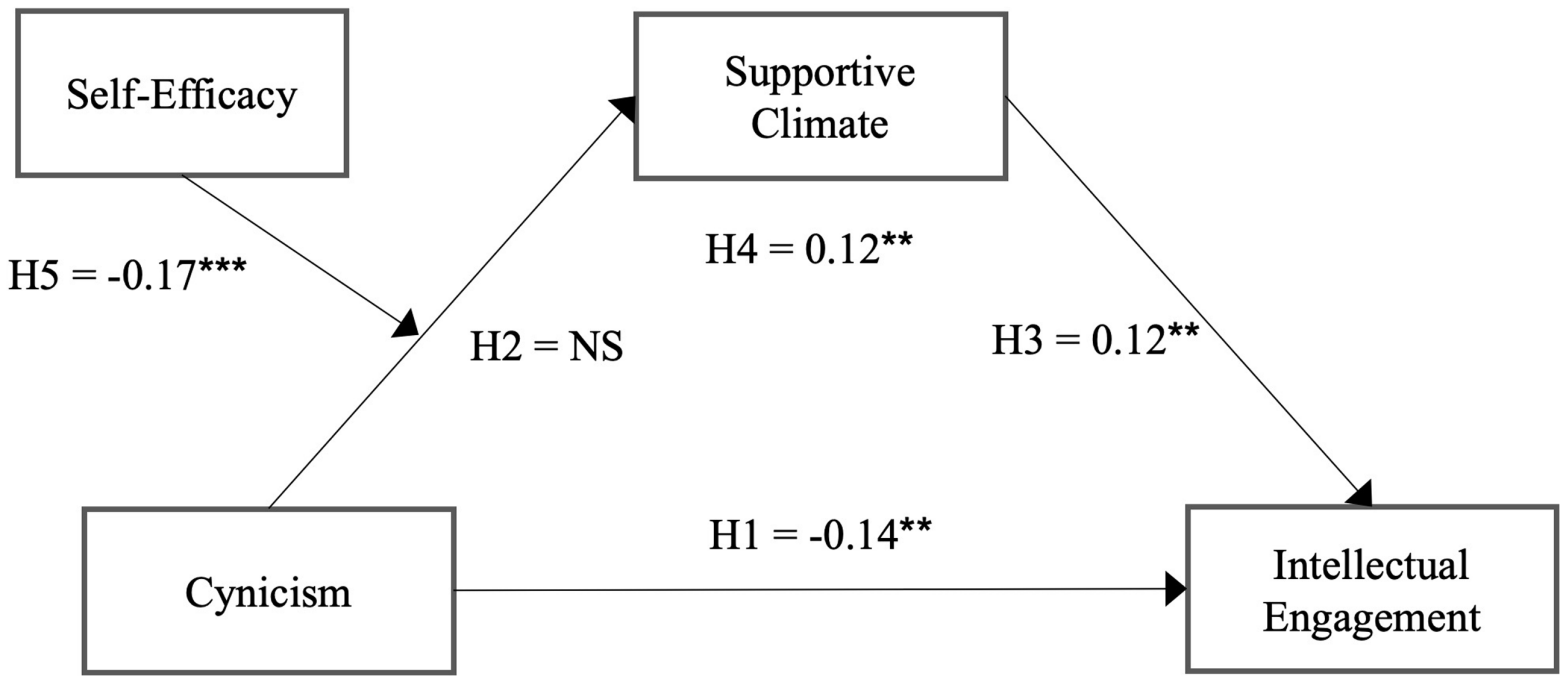
Moderated Mediation Model. The path from cynicism to supportive climate did not reach conventional statistical significance (*p* = 0.054); however, the bootstrapped indirect effect through supportive climate was statistically significant (*b* = −0.020, 95% CI [−0.047, −0.002]), with the confidence interval excluding zero, which supports the mediation effect. NS, not significant, **p < 0.01, ***p < 0.001.

A moderated mediation analysis was conducted (see Table 3; Figure 2) to examine whether the relationship between cynicism (X) and intellectual engagement (Y) is mediated by supportive climate (M), and whether this mediation is moderated by the level (low, medium, high) of self-efficacy (W). Although the direct path from cynicism to supportive climate did not reach conventional statistical significance (*β* = 0.448, *p* = 0.054), the bootstrapped indirect effect was statistically significant (*b* = −0.020, Boot SE = 0.011, 95% CI [−0.047, −0.002]). Following Hayes (Hayes, 2022), the significance of the indirect effect constitutes the primary criterion for establishing mediation, and the confidence interval excluding zero supports this conclusion. Cynicism was negatively associated with intellectual engagement (*β* = −0.142, *p* = 0.002). In turn, supportive climate was significantly associated with intellectual engagement (*β* = 0.120, *p* = 0.011).

**Table 3:** Moderated mediation analysis.

Variables	Coefficient	SE	Bootstrapping BC 95% CI		
			*p*	Lower	Upper
Model 1 (Supportive climate)					
Constant	1.118	0.706	0.093	−0.200	2.577
Cynicism → Supportive climate	0.448	0.232	0.054	−0.008	0.905
Self-Efficacy → Supportive climate	0.683	0.147	<0.001	0.393	0.972
X × W	−0.171	0.049	<0.001	−0.269	−0.074
R^2^ = 0.197, *F* = 27.365, *p* < 0.001					
Model 2 (Intellectual engagement)					
Constant	5.432	0.235	<0.001	4.969	5.896
Cynicism → Intellectual engagement	−0.142	0.045	0.002	−0.231	−0.053
Supportive climate → Intellectual engagement	0.120	0.047	0.011	0.027	0.213
*R*^2^ = 0.275, *F* = 13.189, *p* < 0.001					
Index of moderated mediation: = − 0.020, BootSE = 0.011, BootLLCI = −0.047, BootULCI = −0.002					

SE, Standard Error; *p*, *p*-value; BC CI, Bias-Corrected Confidence Interval; *R*^2^, Coefficient of Determination; *F*, *F*-statistic.

Self-efficacy had a significant effect on supportive climate (*β* = 0.683, *p* = 0.001). The interaction between cynicism and self-efficacy was highly significant (*β* = −0.171, *p* < 0.001). The conditional effects of cynicism at different values of self-efficacy varied by level: low (*β* = −0.239, SE = 0.056, *p* < 0.001, 95% CI = [−0.349, −0.128]), medium (*β* = −0.368, SE = 0.048, *p* < 0.001, 95% CI = [−0.462, −0.273]), and high (*β* = −0.491, SE = 0.064, *p* < 0.001, 95% CI = [−0.624, −0.369]). Thus, the negative effect becomes stronger as the level of self-efficacy increases.

The conditional indirect effect of cynicism on intellectual engagement, through supportive climate, was significant at low (*β* = −0.028, SE = 0.013, 95% CI = [−0.057, −0.005]), medium (*β* = −0.044, SE = 0.019, 95% CI = [−0.084, −0.008]), and high (*β* = −0.060, SE = 0.026, 95% CI = [−0.117, −0.011]) levels of self-efficacy. This effect becomes stronger as the level of self-efficacy increases. The moderated mediation index was significant, confirming that the intensity of the indirect effect of cynicism on intellectual engagement through supportive climate depends on self-efficacy levels. The interaction explained an additional 2.9% of variance (ΔR² = 0.029). Although the moderated mediation index is small (index = −0.020, 95% CI [−.047, −0.002]), the confidence interval excludes zero, supporting the statistical significance of the effect. Small effect sizes are common and theoretically meaningful in organizational research with complex moderated mediation models (Hayes, 2022).

It is important to note that the positive regression coefficient for the path from cynicism to supportive climate (β = 0.448, p = 0.054) should be interpreted in light of the moderated nature of the model. When a moderator is introduced, the regression coefficient for the predictor represents its conditional effect at the mean value of the moderator, not its average marginal effect across all levels. The Johnson–Neyman analysis (Table 4) illustrates this: the effect of cynicism on supportive climate is positive at low levels of self-efficacy and becomes increasingly negative as self-efficacy increases, which is consistent with the observed negative bivariate correlation (*r* = −0.39, *p* < 0.001).

**Figure 3:**
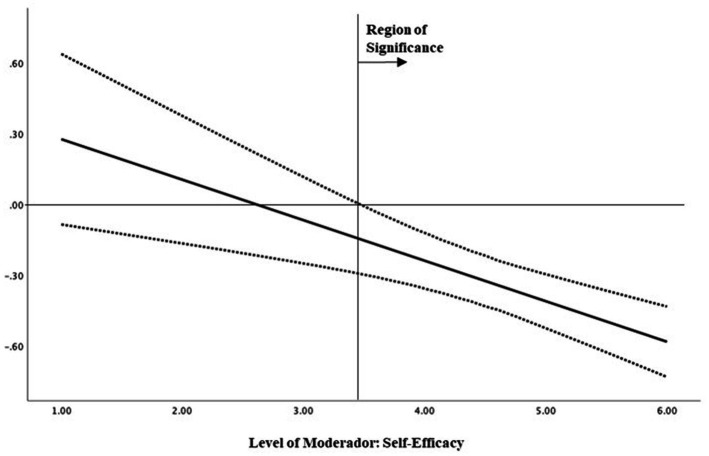
Conditional indirect effect of cynicism on intellectual engagement through supportive climate as a function of self-efficacy.

Finally, Table 4 and Figure 3 present an analysis based on the Johnson–Neyman technique to provide a detailed visualization of the indirect effect of cynicism on intellectual engagement through supportive climate as a function of different self-efficacy scores. The results indicate that the region of significance is between 3.50 and 6.00.

**Table 4:** Conditional effect of focal predictor at values of the moderator.

Johnson–neyman technique						
Self-efficacy scores	Coefficient	SE	T	*p*	Bootstrapping BC 95% CI	
					Lower	Upper
1.00	0.28	0.18	1.50	0.13	−0.86	0.64
1.25	0.23	0.17	1.35	0.18	−0.11	0.57
1.50	0.19	0.16	1.19	0.24	−0.13	0.51
1.75	0.15	0.15	0.99	0.32	−0.15	0.44
2.00	0.10	0.14	0.76	0.45	−0.17	0.37
2.25	0.06	0.13	0.49	0.62	−0.19	0.31
2.50	0.02	0.11	0.16	0.87	−0.21	0.24
2.75	−0.02	0.10	−0.24	0.81	−0.23	0.18
3.00	−0.07	0.09	−0.73	0.47	−0.25	0.11
3.25	−0.11	0.08	−1.34	0.18	−0.27	0.05
3.46	−0.15	0.07	−1.97	0.05	−0.29	0.00
3.50	−0.15	0.07	−2.12	0.04	−0.30	−0.01
3.75	−0.20	0.06	−3.09	<0.001	−0.32	−0.07
4.00	−0.24	0.06	−4.26	<0.001	−0.35	−0.13
4.25	−0.28	0.05	−5.57	<0.001	−0.38	−0.18
4.50	−0.33	0.05	−6.81	<0.001	−0.42	−0.23
4.75	−0.37	0.05	−7.67	<0.001	−0.46	−0.27
5.00	−0.41	0.05	−8.02	<0.001	−0.51	−0.31
5.25	−0.45	0.06	−7.96	<0.001	−0.57	−0.34
5.50	−0.50	0.06	−7.68	<0.001	−0.62	−0.37
5.75	−0.54	0.07	−7.33	<0.001	−0.69	−0.40
6.00	−0.58	0.08	−6.98	<0.001	−0.75	−0.42

SE, Standard Error; T, *T*-statistic; *p*, *p*-value; BC CI, bias-corrected confidence interval.

## Discussion

This research examines the influence of cynicism on intellectual engagement through the mediation of supportive climate and the moderation of self-efficacy. The results allow for the testing of the proposed hypotheses. It was found that cynicism negatively predicts workers’ intellectual engagement; thus, Hypothesis 1 is accepted. This finding is consistent with a recent study (Lehtiniemi et al., 2024). In contrast, Hypothesis 2 is rejected, since the relationship between cynicism and supportive climate was not significant, unlike in a previous study that reported a statistically significant relationship between these two variables (Sen et al., 2022).

Regarding Hypothesis 3, it is accepted, as supportive climate positively predicted intellectual engagement, which is consistent with other research findings (Torrente et al., 2012; Li et al., 2024; Ren et al., 2024; Murray et al., 2025). In addition, the relationship between cynicism and intellectual engagement was found to be mediated by perceived supportive climate; therefore, Hypothesis 4 is accepted, as the bootstrapped indirect effect of cynicism on intellectual engagement through perceived supportive climate was statistically significant (index = −0.020, 95% CI [−0.047, −0.002]). Other studies have also found that a perceived supportive climate functions as a mediating variable (Shi et al., 2022; Tian et al., 2025). In the present study, although statistically significant, the moderated mediation effect was relatively small in magnitude and should therefore be interpreted with caution. Notably, although H2 was rejected, the acceptance of H4 is possible. Following mediation frameworks (Hayes, 2022), the significance of the indirect effect, rather than the significance of each individual path, constitutes the primary criterion for establishing mediation.

Finally, Hypothesis 5, which proposed that the relationship between cynicism and supportive climate was moderated by self-efficacy, is accepted. It is important to note that, although there is a moderating role of self-efficacy, it occurs in a negative direction. Therefore, the effect of cynicism on supportive climate decreases as self-efficacy increases, in other words, the higher the perception of self-efficacy, the more negative the relationship between cynicism and supportive climate. In the proposed model, this means that when self-efficacy is low, cynicism tends to be associated with a greater perception of support, but when self-efficacy is high, it negatively affects the perception of supportive climate.

Thus, the mediation of supportive climate between cynicism and intellectual engagement is conditioned by self-efficacy. A possible explanation for this unexpected finding is that workers with higher self-efficacy may hold higher expectations regarding social and organizational support, and when these expectations are not met, a discrepancy arises between their expectations and perceived reality, which may lead to a more critical perception of supportive climate.

This becomes particularly relevant given that self-efficacy has been considered central in social cognitive theory (Bandura, 1994), conservation of resources theory (Hobfoll et al., 2018), the healthy and resilient organizations model (Salanova et al., 2012a) and the job demands–resources theory (Bakker and Demerouti, 2016). For example, the JD-R theory states that personal resources, such as self-efficacy, are reciprocally related to job resources, such as support (Bakker et al., 2023). From this perspective, personal resources are expected to buffer the impact of demands on well-being through the motivational process (Xanthopoulou et al., 2007); however, personal resources do not always fulfill this protective role (Chen and Hsieh, 2025). In chronically adverse perceived supportive climates characterized by cynicism, personal resources may progressively lose their protective potential, a possibility that, while theoretically plausible, warrants empirical examination in future research. Consequently, when the perceived supportive climate is characterized by cynicism or insufficient support, the gap between expected and available resources becomes more salient and cognitively dissonant for individuals with high self-efficacy than for those with lower levels.

In this study, self-efficacy does not appear to function as a protective resource in this context; on the contrary, it acts as a vulnerability factor, since workers with high self-efficacy perceive more severely the deterioration of supportive climate associated with cynicism, which ultimately negatively impacts intellectual engagement. In contrast, empirical evidence has widely supported the protective role of self-efficacy (Bu et al., 2021; Edokpolor et al., 2024).

Additionally, from conservation of resources perspective (Hobfoll et al., 2018), when self-efficacious individuals invest their personal resources to compensate for a deficient perceived supportive climate, resource depletion may trigger loss spirals that further reinforce cynical attitudes. The present study adds to a growing body of evidence documenting the negative consequences of self-efficacy in workplace contexts (Del Líbano et al., 2012; Salanova et al., 2012b; Vancouver et al., 2014), highlighting that self-efficacy does not always act as a buffer (Islam et al., 2021). For example, self-efficacy may have curvilinear consequences on employee effort, such that excessively high levels can paradoxically reduce effort and lead to negative outcomes (Bachrach et al., 2023).

At this point, it is important to note that not all resources are effective in coping with various types of demands in the work context (Shu-Ling et al., 2018). Thus, increasing all job resources does not necessarily produce positive results; an integral reading of personal resources requires considering both individual and contextual characteristics (Salanova et al., 2012b; Díaz-Fúnez et al., 2024).

This phenomenon aligns with what some authors in the field of business strategy have referred to as *paradoxical resource trajectories* (Miller and Le Breton-Miller, 2021). In our study, self-efficacy behaves similarly: in the presence of cynicism and low perceived support, it does not act as a buffer but rather as a vulnerability factor. Based on these findings, and in an exploratory manner, we tentatively propose referring to this phenomenon as “the resources paradox.” This exploratory finding suggests that, in the presence of negative states (e.g., cynicism), a high level of personal resources (e.g., self-efficacy) may become counterproductive for job and collective resources (e.g., supportive climate).

In fact, negative effects of self-efficacy have been documented, particularly in generating overconfidence, which increases the likelihood of errors in analytical tasks (Vancouver et al., 2002) and is associated with demotivation and insufficient learning capabilities (Lu et al., 2022). Ultimately, self-efficacy in high doses, without a supportive environment, may reveal its “dark side.” Therefore, self-efficacy requires a context that facilitates its development, which implies a work team free from cynical behaviors and characterized by social support practices.

### Theoretical and practical implications

From a theoretical standpoint, the findings of this study suggest that it is not enough to promote self-efficacy without simultaneously strengthening the organizational support context. Without doing so, those with greater personal resources (self-efficacy) could paradoxically be the ones experiencing greater distress in the organizational context. These results challenge the traditional view that assumes self-efficacy as a protective resource. The above could contribute to enriching the Job Demands–Resources theory.

Given that the sample is predominantly composed of workers from the correctional, healthcare, and educational sectors in Colombia, the following practical implications should be interpreted within this specific organizational context. At a practical level, before designing a program aimed at enhancing workers’ resources, organizations must consider the idiosyncrasies of their teams, as well as their resources and interests, which is vital to avoid generating adverse effects (Díaz-Fúnez et al., 2024). This highlights the importance of not assuming that well-being depends solely on individual characteristics; rather, organizations and teams must create conditions that enable workers’ resources to be developed. In this regard, organizational interventions should be ecological, multilevel, and context-specific, rather than merely individual.

### Limitations and future research directions

At least five limitations can be identified in this study: (1) the use of self-report questionnaires, which may be associated with social desirability and various cognitive biases; (2) the measurement of all variables using the same instruments at a single time point, which introduces the risk of common method bias; (3) the non-probabilistic sampling strategy, which limits the representativeness of the sample and may introduce selection bias; (4) the cross-sectional design, which precludes causal inference and limits the interpretation of reported relationships as predictive associations rather than causal influences; (5) the sample composed of workers from different regions of Colombia, with a strong concentration of employees from prisons, healthcare institutions, and educational institutions, which limits the generalization of these findings to other occupational groups and cultural contexts.

Based on the above, it is recommended to include outcome variables, such as job performance or turnover, as well as to conduct longitudinal studies that allow capturing the evolution of the behavior of the variables at different points in time, and to adopt a cross-cultural approach to determine whether the results proposed here differ across cultures. In addition, it would be interesting to study the psychological phenomena that explain why self-efficacy may act as a vulnerability factor in certain organizational contexts, which would contribute to understanding the paradox of resources. It would also be valuable to examine whether the same occurs with other personal resources, such as optimism or resilience.

## Conclusion

Cynicism directly reduces intellectual engagement, but it also does so indirectly through supportive climate. The moderated mediation index was significant, confirming that the intensity of the indirect effect of cynicism on intellectual engagement through supportive climate depends on self-efficacy levels. It is concluded that, as self-efficacy increases, the effect of cynicism on intellectual engagement through perceived supportive climate becomes more negative. This evidence suggests that, in this context, self-efficacy does not operate as a protective resource; on the contrary, it appears to amplify the negative effects of cynicism on workers’ perceptions of supportive climate. In other words, not all personal resources are beneficial in all contexts.

## Data Availability

The raw data supporting the conclusions of this article will be made available by the authors, without undue reservation.
